# Using Citizen Science to Address Out-of-Pocket Healthcare Expenditure with Aboriginal Communities in the Far West of South Australia: A Protocol

**DOI:** 10.3390/ijerph22111640

**Published:** 2025-10-28

**Authors:** Courtney Ryder, Ray Mahoney, Patrick Sharpe, Georga Sallows, Karla Canuto, Andrew Goodman, Julieann Coombes, Odette Pearson, Jaquelyne T. Hughes, Marlien Varnfield, Candice Oster, Jonathan Karnon, Claire Drummond, James A. Smith, Shanti Omodei-James, Lavender Otieno, Ali Soltani, Billie Bonevski

**Affiliations:** 1 Flinders Health and Medical Research Institute, College of Medicine and Public Health, Flinders University, Kaurna Yerta, Adelaide 5000, Australia; 2 The George Institute for Global Health, University of New South Wales, Gadigal, Sydney 2000, Australia; 3 School of Population Health, University of New South Wales, Gadigal, Sydney 2000, Australia; 4 Commonwealth Scientific Industrial Research Organisations, Turrbal and Yuggera, Brisbane 4000, Australia; 5 Far West Community Partnerships, Kokatha, Mirning and Wirangu Country, Ceduna 5690, Australia; 6 Wardliparingga, South Australian Health and Medical Research Institute, Kaurna Yerta, Adelaide 5000, Australia; 7 Faculty of Health and Medical Sciences, University of Adelaide, Kaurna Yerta, Adeliade 5000, Australia; 8 College of Nursing and Health Sciences, Flinders University, Kaurna Yerta, Adelaide 5000, Australia; 9 Caring Futures Institute, Flinders University, Kaurna Yerta, Adelaide 5000, Australia; 10 School of Medicine and Public Health, The University of Newcastle, Awabakal, Newcastle 2267, Australia

**Keywords:** out-of-pocket health expenditure, citizen science, Web App, mixed methods, community engagement, Aboriginal and Torres Strait Islander

## Abstract

Out-of-pocket health expenditure (OOPHE) significantly impacts people with chronic and complex diseases (CCDs) and injuries. Aboriginal communities experience a higher burden of CCDs and injury, along with greater OOPHE inequities. This project aims to develop and implement a social prescribing digital platform (Web App) to reduce OOPHE. It is grounded in citizen science approaches that value the lived experience and knowledge of Aboriginal people in shaping solutions. The project uses a citizen science methodology adapted for these communities, using knowledge interface methodology to weave together Indigenous and Western knowledges. Research methods (Indigenous, quantitative, qualitative) explore the relational nature of OOPHE risks and protective factors through co-design and workshops with Aboriginal participants to develop the Web App. A community-centric developmental evaluation guides the trial and refinement of the platform, allowing for ongoing learning and adaptation. Process measures inform a national scale-up and evaluation framework. Addressing OOPHE is essential to improving health and wellbeing for Aboriginal and Torres Strait Islander individuals and families living with or at risk of CCDs. This initiative aims to reduce the impact of OOPHE through digital social prescribing, there by connecting people with essential community services to access healthcare, offering a scalable approach to addressing health inequities nationwide.

## 1. Introduction

Australia is facing a major political crisis, branded as the ‘cost-of-living crisis’, resulting from precarious economic stability [1]. At the center of this crisis are Australian households, affordability challenges, and the rising cost of food, transport, energy and housing [1]. This crisis includes out-of-pocket healthcare expenditure (OOPHE), which is often invisible in this debate but relates to additional expenses (i.e., travel for medical appointments, medication, equipment, and lost income from taking time off work) not covered by Australia’s universal taxpayer-funded health insurance (i.e., Medicare) or Private Health Insurance [2,3]. It is estimated that one in four Australians will forgo medical treatment due to OOPHE burden [4]. Catastrophic OOPHE can induce bankruptcy [3], with ill health from OOPHE one of the top four cited reasons for personal insolvency in Australia [5]. It is estimated the economic burden over 2019–20 from OOPHE was AUD 30 billion, this included costs associated with delaying treatment or not filling prescriptions [3,6].

Out-of-pocket healthcare expenditure burden is dictated by a social gradient, through the way in which the social, commercial and cultural determinants of health interact, which give rise to OOPHE risk and protective factors [3]. In this protocol Aboriginal people refers to the First Peoples of mainland Australia, while Torres Strait Islander people refers to the First Peoples of the Torres Strait Islands, the term ‘Indigenous knowledges’ respectfully acknowledges global Indigenous knowledges that have existed since time immemorial. Burden of OOPHE is also exacerbated by greater chronic disease and injury profiles in Australian households [3]. The last Australian OOPHE senate enquiry identified Aboriginal and Torres Strait Islander households as the most vulnerable to OOPHE impacts and burden [3]. This can be observed through the disproportionate burden of chronic and complex diseases (CCD) and injuries caused through ongoing colonization: with greater rates of asthma (1.6 times); emphysema (4.4 times); chronic obstructive pulmonary disease (3 times); metabolic diseases (3.7 times); diabetes (2.6 times); hospitalized intentional self-harm (3 times); and death from injury or poisoning (2.1 times) than that of the dominant (non-Indigenous) population [7,8]. These CCDs and injuries have the greatest OOPHE impacts in Australia [4].

Aboriginal families have reported using a variety of coping mechanisms at times of high to catastrophic levels of OOPHE burden, such as deferring GP visits [3], avoiding medical treatment, sleeping rough, and borrowing money [2,7]. Specific to chronic kidney disease, Aboriginal patients have forgone dialysis treatment, resulting in death [9]. All of these coping mechanisms come at a significant ongoing physical and emotional cost, which is further exacerbated by colonization, for Aboriginal families and communities [2,7]. Previous research by this team with Aboriginal communities in the Far West of South Australia (SA) [7] and families impacted by burns injuries [2] has found that family and community connections, as well as social, cultural and emotional determinants, are important protective factors for mitigating OOPHE, whereas risk factors include government income management or an inability to fully participate in the workforce due to health issues, which significantly reduces household income [7]. It is through this work that the Far West community has urgently called for effective community-centric solutions that directly address OOPHE risk factors for their community, particularly patients with chronic and complex diseases or injury, to improve health equity and outcomes in the Far West region [2].

### 1.1. Social Prescribing to Address OOPHE

Internationally social prescribing is described as:

“a holistic, person centred and community-based approach to health and well-being that bridges the gap between clinical and nonclinical supports and services. By drawing on the central tenets of health promotion and disease prevention, it offers a way to mitigate the impacts of adverse social determinants of health and health inequities by addressing nonmedical, health-related social needs... it is recognised as being a means for trusted individuals in clinical and community settings to identify that a person has nonmedical, health-related social needs and to subsequently connect them to nonclinical supports and services within the community.”

([10], page 9)

It presents as a potential solution for addressing OOPHE, through providing patients with targeted support to access healthcare, which can include fuel vouchers or affordable accommodation close to hospitals. The literature suggests that the overall social prescribing process should have two phases (planning and implementation), with each containing six processes [11] pages 9–12:

Planning Phase: Identify the following: 1. target population, 2. non-medical needs to address, 3. available supports and services, 4. delivery site, 5. staffing, 6. funding

Implementation Phase: 1. participant identification, 2. need for support worker, 3. non-medical screening, 4. non-medical referrals (services and supports), 5. additional supports needed, and 6. follow up.

In Australia, social prescribing aligns well to funding and policy frameworks, with the Royal Australian College of General Practitioners (RACGP) and Consumer Health Forum highlighting the critical need for locally implemented and nationally scalable approaches to social prescribing [8,12]. Both the National Aboriginal Community Controlled Health Organisation (NACCHO) [13], and the National Preventive Health Strategy recommend social prescribing to address health system challenges at the local level [14]. Furthermore, the World Health Organization (WHO) acknowledges that social prescribing can benefit those experiencing “the greatest inequity and vulnerability to poorer health outcomes” ([12], page 5).

Social prescribing in Australia, however, is still considered to be in a state of infancy. Additionally, evidence on effectiveness is still emerging internationally. Despite this, social prescribing interventions continue to be widely used in the United Kingdom and United States, demonstrating positive effects on self-efficacy, quality of life, mental health, wellbeing, loneliness, and healthcare utilization. This includes being used to target social determinants of health, including financial burden and socioeconomic deprivation [15,16]. Social prescribing interventions are effective in addressing social determinants of health by connecting individuals and families with voluntary and community services for health, wellbeing, and social care. For example, evaluated CCD social prescribing programs have increased patient self-efficacy through access to voluntary and community services, as well as decreasing unplanned hospital admissions [16]. In Australia, social prescribing for occupational injury has demonstrated improvements in individual biopsychosocial wellbeing, reduced health service use, and increased confidence and frequency relating to participating in work and social activities [17]; for mental illness, it has improved quality of life and overall health status [18].

This protocol defines the scope of a study designed to address a key priority identified by Aboriginal leaders in the Far West region of South Australia (SA), in terms of understanding and addressing the impacts of OOPHE for Aboriginal families. The protocol encompasses the co-design, implementation, and evaluation of an OOPHE social prescribing Web App, with a focus on community-led approaches and culturally relevant solutions.

### 1.2. Aims

The study aims to co-design a social prescribing digital platform (Web App) for OOPHE, underpinned by citizen science approaches to harnessing the wealth of knowledge amongst consumers in Aboriginal and Torres Strait Islander communities with lived experience of and passion for creating and determining solutions. The specific aims of this project are:

Aim 1: Co-create new knowledge with citizen scientists on risk and protective factors to OOPHE, which support or inhibit access to healthcare for Aboriginal communities in rural and remote regions.

Aim 2: Co-develop and evaluate an Aboriginal OOPHE social prescribing Web App, connecting Aboriginal families and health professionals to support initiatives through social prescribing, to increase healthcare access and health and wellbeing outcomes.

## 2. Materials and Methods

**Figure 1:**
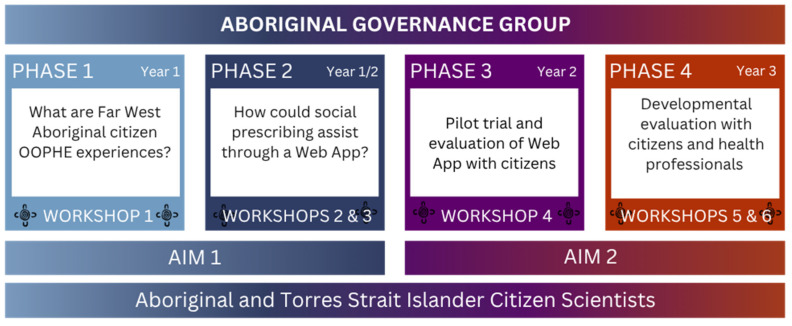
Study overview.

This is an Aboriginal-led and focused mixed-method (Indigenous, quantitative and qualitative) co-design implementation and evaluation study, with four phases that employ a citizen science methodology, as illustrated in Figure 1: Phase 1 Co-creation of OOPHE Knowledge, Phase 2 Web App Co-design, Phase 3 Web App Pilot, and Phase 4 Web App Evaluation. This study builds on a strong foundation of Indigenous knowledges and voice in the Far West, focused on OOPHE experiences.

### 2.1. Methodology

This Aboriginal-led study, with a collection of Aboriginal, Torres Strait Islander and non-Indigenous researchers, collaboratively adapted the citizen science methodology to develop and evaluate an OOPHE social prescribing Web App. This includes the application of a knowledge interface methodology to weave together Indigenous and Western research knowledge systems and methodologies [19]. Through this process, knowledge systems, research methodologies, and methods are integrated through mutual respect, shared benefits, human dignity, and discovery for new knowledge formation [19]. In this process, power differentials are redressed for Indigenous and non-Indigenous knowledges [19]. This methodology is used across the four phases of the study.

### 2.2. Citizen Sciences for Community-Centric Research

The historical nature of citizen scientists, from a Western centric paradigm, can be traced back to the 17th and 18th centuries through naturalists and public observers [20]. However, for Aboriginal communities, Australia’s first researchers, citizen science reflects a continuation of knowledge that has existed since time immemorial, grounded in relational, place-based, and intergenerational practices of inquiry and stewardship [19,21,22,23]. In the context of social prescribing, best practice research with Aboriginal and Torres Strait Islander communities requires “co-design with all relevant stakeholders” ([20], page 3). The citizen science methodology ensures centrality in this process, through increasing community understanding and involvement in research, and in ensuring research addresses the community’s health and wellbeing requirements, particularly when integrated with Indigenous research principles of respect, reciprocity, relationality, and responsibility [13,24,25,26]. This will be enacted throughout all study processes; design, implementation, evaluation, and outcome translation. Citizen scientists, who are members of the Far West Aboriginal community, will play a central role in the co-design, co-production, participant recruitment, and data collection for an OOPHE initiative that meets individual and community needs.

### 2.3. Setting

This project is conducted in the Far West of SA. Kokatha, Mirning, Wirangu, Pitjantjatjara country covers the Far West over the townships of Ceduna and Aboriginal communities of Yalata, Scotdesco, Kooniba and Oak Valley. An estimated 1139 Aboriginal people live in this region, where displacement from colonization has resulted in a range of Aboriginal language groups being represented [27]. Across the Far West, hospital separations for six priority chronic and complex diseases (cardiovascular, diabetes, cancer, oral health, kidney, and respiratory disease) range from 36 to 195 per 1000 people per year [27].

### 2.4. Participants

Participants with lived experience take part as citizen scientists and as research participants (Phases 1–4). The inclusion criteria require participants identify as an Aboriginal and/or Torres Strait Islander person, aged >18 years, having or caring for a family member with a chronic and complex disease(s) or injury requiring ongoing treatment, and able to provide informed written and verbal consent. Health professional participants (Phase 3) are aged >18 years and work with Aboriginal patients and communities in the Far West. Participants who fall outside of these eligibility requirements will not be permitted to participate in any phases of the study.

### 2.5. Citizen Science Governance

Community-centric research will be further enacted through the establishment of an Aboriginal Governance Group (AGOV) which will be made up of Chairpersons and Chief Executive Officers of the local surrounding Aboriginal communities from the Far West (Ceduna, Yalata, Scotdesco, Koonibba, and Oak Valley), along with Aboriginal investigators. Continuing project oversight by AGOV will ensure strong community partnerships and collaborations underpin conduct of the study, facilitate community input, and provide high levels of oversight of methods and relevance, along with input into outcome translation, community data repatriation at the end of the project and ownership and responsibility of the Web App going forward. The AGOV members will have autonomy in selecting the Aboriginal-community-based researchers and citizen scientists participating in each phase of the project. Throughout this project, there will be a two-way learning process, with AGOV, citizen scientists and community researchers educating the investigation team on their needs and experiences of OOPHE. Research training will be provided as part of capacity building surrounding tertiary institute research knowledge and experience.

### 2.6. Ethics

Ethical approval was obtained from the S Aboriginal Health and Research Ethics Committee in (04-24-1166), Flinders University Human Research Ethics Committee (0473), and the Commonwealth Scientific and Industrial Research Organisation (CSIRO) Health and Medical Human Research Ethics Committee (2025_017_RR).

### 2.7. Phase 1: Co-Creation of OOPHE Knowledge

Phase 1 of the project aims to co-create new knowledge with citizen scientists on OOPHE risk and protective factors that support the prevention of OOHPE and improve access to healthcare within Aboriginal and Torres Strait Islander communities. This phase will be initiated through the establishment of a citizen science representative group, consisting of two community-based lead researchers (termed Community Navigators) and 10 citizen scientists who are lived-experience OOPHE participants from the Far West. Community Navigators will be recruited and vetted through Far West Community Partnerships (FWCP); both Community Navigators and FWCP will recruit citizen scientists. Citizen scientists and Community Navigators will be provided with formal training regarding Indigenous research methodologies, participatory developmental evaluation and cross-cultural co-design through programs run by the Australian Centre for Social Innovation and Lowitja Institute. They will play a central role throughout the project in co-creating OOPHE knowledge, developing initiatives and conducting evaluations, with support from the investigation team.

Knowledge of OOPHE lived experience will be co-created to understand patient and family journeys for co-design workshops. Community Navigators will recruit 10 lived-experience participants using purposive sampling for yarning sessions. The Indigenous research method of Yarning, which follows a prescribed conversational technique around key themes, will be used for rich data collection [28]. Yarning sessions will be conducted on Country by Community Navigators with shadowing support from members of the investigation team, to explore OOPHE experiences along with social and cultural risk and protective factors. This shadowing will include a trainer approach, where an investigation team member will support the Community Navigator in conducting yarns, until they feel comfortable to do this by themselves. All yarns will be transcribed and thematically coded by Community Navigators and members of the investigation team. This will focus on a collaborative approach for theme identification, to ensure that outcomes focus on the lived experience and knowledge of Far West communities [7].

Mapping the lived experience of OOPHE impacts for Far West families and patients will occur during Workshop 1, on Country, with citizen scientists and AGOV members. These workshops will be led by Community Navigators and supported by the investigation team. During Workshop 1, a first-level thematic analysis of yarning sessions will be presented for further analysis and patient journey mapping. Community navigators will then work with investigators to explore similarities and differences across patient journeys for the creation of experience narratives for the next phase of the project. Outcome translation for Aim 1 will include two video resources capturing OOPHE journeys. These videos will focus on assisting users to understand OOPHE, and to reduce stigma, burden and shame from OOPHE.

### 2.8. Phase 2: Web App Co-Design

Phase 2 aims to develop an Aboriginal and Torres Strait Islander co-designed OOPHE social prescribing Web App. This App will connect families and health professionals to community initiatives, increasing healthcare access and improving health and wellbeing outcomes. Phase 2 primarily focuses on the co-design aspects of the social prescribing Web App, with service mapping to culturally safe and responsive options to reduce OOPHE burden.

Co-design will occur over two workshops, led by community navigators and research team members, with the first conducted to align the OOPHE patient journey and risk and protective factors to social prescribing pathways, identified in Phase 1. For this workshop, social prescribing co-design workbooks will be culturally adapted from prior research conducted by the investigators [29]. Workshop participants will include citizen scientists and members of the AGOV. A range of mediums (i.e., PowerPoint, story board) will be used to present OOPHE patient journey data and information on social prescribing. Participants will use the adapted workbooks to express their preferences for different components of social prescribing. Throughout the workshop, participants will create, share and refine their ideas for the social prescribing Web App. The best-practice framework for culturally safe eHealth interventions with Aboriginal and Torres Strait Islander peoples will be applied throughout this process [30], when designing the Beta Version of the Web App. The Beta Version will include co-designed mapping content and processes from workshop 2, along with functionality for users to post information about resources, services or lived experiences.

Service mapping will be undertaken during Workshop 2 and 3 by Community Navigators, supported by the investigator team using a cultural safety framework [31], to determine and decide which local, regional, state and national organizations are culturally responsive for inclusion in the Web App. This service mapping will be conducted by the investigation team and will include an audit of service provision available to Aboriginal and Torres Strait Islander patients across South Australia. The Beta Version of the Web App will incorporate the identified services, along with translation videos from Phase 1 and a psychometrically assessed OOPHE survey tailored for Aboriginal communities, to identify needs and signpost to local services. The Beta Version of the Web App will be presented in the third workshop for discussion and the further development, refinement and creation of a Web App implementation strategy. Cooley and Kohl’s Scalability Assessment Tool will be used to assess Web App scalability during this period [32].

### 2.9. Phase 3: Web App Pilot

Following approval of the Beta Version, the Web App will be trialed over Phase 3 of the study. A mixed-methods approach will be used to assess the usability and acceptability of the Web App, with a sample of 20 participants: 15 Aboriginal participants with lived experience of OOPHE and 5 healthcare workers. Recruitment of participants will take place through citizen scientists and engagement with Far West health and community services. These participants will be asked to engage with the Web App over a one-month period and complete a survey exploring the system usability of the App [33]. Two group yarning sessions (one with Aboriginal participants and one with health professionals) will explore views on the acceptability and usability of the Web App. Yarning sessions will be conducted and thematically analyzed by Community Navigators on Country, with support from the investigation team. Outcomes related to the usability and acceptability of the Web App will be presented at an implementation workshop (Workshop 4) with citizen scientists and AGOV members to inform refinement and development of the final Web App, as well as to develop an implementation strategy for the sustainability and scalability of the Web App.

### 2.10. Phase 4: Web App Evaluation

**Figure 2:**
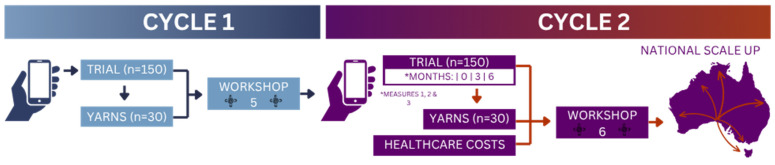
Two cycles of developmental evaluation.

Developmental evaluation—a participatory method that generates feedback to inform adaptation of innovations to their operating environments over “repeated cycles of data collection, feedback, reflection and adaptation.” [34], page 2—will be employed. This will be citizen supported over two cycles, with 150 Aboriginal individuals with a chronic and complex disease or injury evaluating the App’s implementation (Figure 2). This sample size has been calculated based on 2.4% (43,000) Aboriginal individuals residing in SA, using $n=\frac{Z^{2}p(1-p)}{e^{2}}$ with an 8% margin of error (*e*), 1.96 *Z-score* (95% confidence interval) and an estimated proportion of 0.5 (*p*) [35]. The primary outcome during the developmental evaluation will focus on the impacts of OOPHE, measured using a culturally validated OOPHE tool developed by the research team [7].

During the first cycle, the Web App will be implemented with participants across South Australia (SA) over a three-month period. Recruitment will occur through social media and community newsletters, facilitated by QR codes. During this period, data will be collected on the Web App’s implementation processes, uptake, and usage, to explore the conditions necessary for successful implementation and scale-up. Community Navigators will conduct yarning sessions with 30 participants, exploring their experiences using the Web App. Data will be co-analyzed descriptively by Community Navigators, with support from the investigation team, and presented at Workshop 5 with citizen scientists and AGOV members, to determine the tailoring needs of the Web App for further refinement prior to cycle two.

In cycle two, the Web App will be implemented across SA, following the same recruitment process of cycle one. The following data will be collected for outcomes at baseline, three, and six months during cycle two:

1. Out-of-Pocket Health Expenses (OOPHE), impacts, and associated cultural and social determinants (OOPHE survey) (Primary Outcome)
2. Self-management of chronic and complex diseases and injuries (Partners in Health Scale, a chronic condition self-management scale validated in Aboriginal communities)
3. Health-Related Quality of Life (assessed using the EQ-5D-5L scale)

Web App participants’ healthcare use and costs six months prior to baseline through to six months post-baseline will be accessed through Australia’s universal taxpayer-funded health insurance subsidy schemes (Pharmaceutical Benefits and Medical Benefits Schemes). Outcome analysis will be conducted using R software version 4.5.0. Descriptive statistics will be used to describe baseline characteristics of participants. The difference in means for outcome measures will be evaluated over repeated timepoints using analysis of variance. Significance of timepoints and outcomes will be assessed using ANOVA, where a 95% confidence interval will be reported with a significance level of *p* < 0.05 [36]. Interrupted time-series analyses of healthcare use and costs will be undertaken to estimate the effect of the Web App on the use of healthcare, adjusting for individual trends in healthcare use prior to the implementation of the Web App [37]. The outputs from these analyses will be presented as a cost consequence analysis. Yarning sessions will also be conducted by Community Navigators with 30 participants during cycle two, focusing on Web App implementation processes, uptake, and usability. This data will be collected and analyzed descriptively. A blended hybrid approach combining quantitative data and qualitative outcomes from yarning outcomes will be used for evaluation of the Web App against the primary outcome of health service utilization, which will be shaped through the lived-narrative of citizen scientists and AGOV members.

### 2.11. National Scale-Up

A national scale-up workshop (Workshop 6) will communicate the implementation and outcome data of the Web App, run by Community Navigators and members of the research team, and supported by citizen scientists and AGOV members. A range of translation partners, government officials and key stakeholders (e.g., primary health networks, local health networks), to address a national approach for translation of the Web App will participate. This workshop will co-develop national scale-up strategies and evaluation plans for the Web App, including economic analysis, interrupted time-series assessment, and a blueprint for reform to mitigate OOPHE for Aboriginal and Torres Strait Islander communities.

### 2.12. Key Research Outcomes and Impact

This Web App aims to increase healthcare access along with health and wellbeing outcomes through connecting Aboriginal and Torres Strait Islander families and health professionals to community initiatives. It will provide connection at both individual and community levels, where Aboriginal and Torres Strait Islander citizens connect and support each other and act to improve healthcare access, literacy, and outcomes. A national blueprint of reform for the Web App will provide a comprehensive guide for translation across primary and local health networks and key Aboriginal community and stakeholder organizations, facilitating national scalability. Finally, this project seeks to empower and involve Aboriginal and Torres Strait Islander citizens in research engagement and processes, ensuring ongoing ownership beyond the life course of this project.

### 2.13. Anticipated Challenges

Knowledge co-creation for a Web App with Aboriginal communities presents unique opportunities for innovation and empowerment, but it is not without limitations and challenges, which requires careful navigation and cultural humility by the research team. These challenges go beyond standard impacts related to the recruitment or retention of citizen scientists and Aboriginal participants, or community business during data collection periods (i.e., sorry business, seasonal cultural activities). They necessitate ongoing collective reflexivity, particularly in ensuring that Web App design is responsive to community-identified priorities, grounded in evidence and culturally safe engagement processes, and authentically usable in ways that reflect Indigenous ways of knowing, being, and doing [26]. We must also acknowledge the inherent limitations and scope of Web App initiatives, ensuring these are clearly articulated throughout the testing, trial and evaluation phases [26].

## 3. Conclusions

Addressing OOPHE and the associated impacts is important for improving health and wellbeing outcomes for Aboriginal patients and their families with, or at risk of developing, CCDs. Aboriginal citizens, in collaboration with Aboriginal, Torres Strait Islander and non-Indigenous researchers, will co-design, implement and evaluate an OOPHE social prescribing Web App. Indigenous knowledges will be central throughout, ensuring that Aboriginal citizens have their lived experience validated, safely shared and integrated, to decrease the burden and stigmatism associated with OOPHE across Aboriginal households. The proposed scalable method aims to tackle health inequalities at a nationwide level.

## Data Availability

The data generated from this study will not be readily available, as they are governed by Indigenous Governance principles for Indigenous Data Sovereignty. In accordance with Indigenous Governance frameworks, data access is restricted to protect cultural knowledge, uphold relational accountability, and ensure that data use aligns with community values and priorities. Requests to access the datasets should be directed to courtney.ryder@flinders.edu.au.

## Abbreviations

The following abbreviations are used in this manuscript:

OOPHE	Out-of-Pocket Healthcare Expenses
CCD	Chronic and Complex Disease
FWCP	Far West Community Partnerships
SA	South Australia
AGOV	Aboriginal Governance Group
NACCHO	National Aboriginal Community Controlled Health Organisation
CSIRO	Commonwealth Scientific and Industrial Research Organisation
